# Taenia saginata: an unusual cause of post-appendectomy faecal fistula

**DOI:** 10.11604/pamj.2016.25.200.11003

**Published:** 2016-11-29

**Authors:** Mohammed Najih, Hicham Laraqui, Nouredine Njoumi, Faycel Mouhafid, Mountassir Moujahid, Abdelkader Ehirchiou, Aziz Zentar

**Affiliations:** 1 Department of Surgery, Mohamed V Military Hospital, Mohamed V, Souissi University, Rabat, Morocco

**Keywords:** Taenia saginata, faecal fistula, post-appendectomy

## Abstract

Post-appendectomy faecal fistula is a rare surgical complication, associated with significant morbidity. Taenia saginata infestation is one of the most common cestode infestation in the gastrointestinal tract. It makes many complications as obstruction, perforation, anastomotic leakage or appendicular stump dehiscence. The objective of our study is to report a very rare case of post appendectomy faecal fistula caused by taenia saginata infestation and was successfully treated conservatively.

## Introduction

Known as the beef tapeworm. Teania saginata infection is reported in all countries where raw or undercooked beef is consumed [1, 2]. Although the tapeworm seems to be a benign parasitic disease, it can cause serious surgical gastrointestinal tract complications [3]. We present here a very rare case of enterocutaneous post appendectomy fistula related to taenia saginata infestation.

## Patient and observation

**Figure 1:**
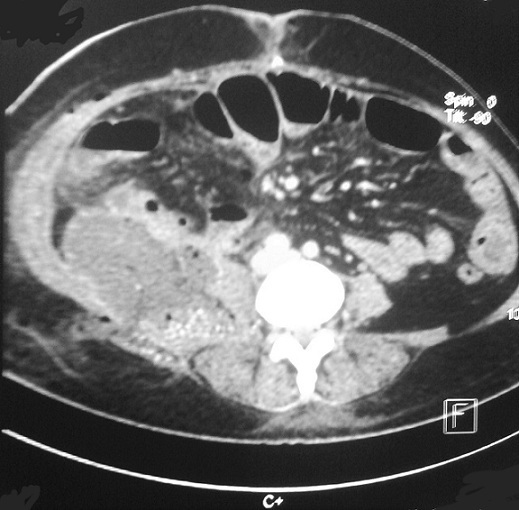
Computed tomography (CT) scan revealed a fluid collection in the psoas, associated with inflammatory changes in the local fat and a small amount of free liquid in the right parietocolic area

**Figure 2:**
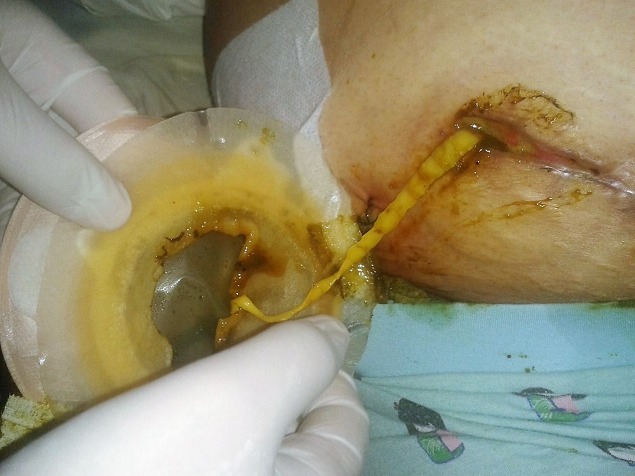
On the third day, an enterocutaneous fistula was observed and a long tapeworm emerged from the McBurney’s incision

**Figure 3:**
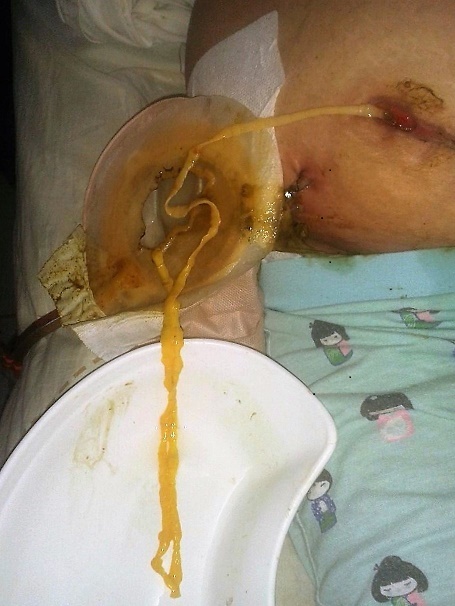
The cestode was removed attentively in a single piece

A 51 year-old women came to the emergency department with right abdominal pain, nausea, vomiting and fever at (38°C). The pain did not radiate to any other site and did not change with various positions. The Physical examination revealed a McBurney's point, without psoas signs. The biological assessment showed leukocytosis at 16200/m^3^. The ultrasound study showed a free fluid in the right lower quadrant with a non-compressible structure over the iliac vessels suggesting an acute appendicitis. An appendectomy was performed by McBurney's incision. The patient was discharged two days later without any complications. Pathology report showed an acute appendicitis with periappendicular reaction. 12 days later, the Patient came back with lump in the right iliac fossa with pain and high grade fever. The physical exam found localized fixed mass in the Right Iliac Fossa. Blood exams revealed 14,000 White Blood Cells with 80% Neutrophiles. A C-reactive protein level was at 112 mg/L. The ultrasonography was showing localized collection in Right Iliac Fossa. Computed tomography (CT) scan revealed a 10cm × 4.7 cm fluid collection in the psoas, associated with inflammatory changes in the local fat and a small amount of free liquid in the right parietocolic area (Figure 1). A Computer tomography (CT) scan- guided percutaneous aspiration of the abscess has been performed and a catheter to drain the abscess was placed. On the third day, an enterocutaneous fistula was observed and a long tapeworm emerged from the McBurney's incision (Figure 2). The cestode was removed attentively in a single piece (Figure 3). The fistula was managed conservatively and the patient was discharged on 15^th^ post-operative day after she received a single dose of niclosamide (4x500 mg). Two weeks later, she returned without any pain or fever. The fistula was dried out and the wound was properly healed without any signs of ongoing infection. The parasitological exam revealed Teania saginata.

## Discussion

The adult stage of Taenia saginata are the intestinal tapeworms for which humans are the only definitive hosts [4]. the adult’s stage are harbored in human intestines, which act as the definitive host, and the larval or cysticercus stages develop in the tissue of cattle. Human behavior is essential for their survival, since contamination of fields with human faeces leads to the infection of the cattle, and the habit of ingesting raw or undercooked meat closes the cycle with the infection of humans. [5]. Once the cysticercus is ingested, the adult worm develops in approximately 2 months. Most infected person is asymptomatic; the usual symptoms are vague or mild abdominal pain or discomfort. Less common symptoms are nausea, change in appetite, weakness and weight loss [2]. The length of adult worm is usually less than 5 m for T. saginata; however, it may reach up to 25 m [6]. The tapeworm attaches to the mucosal surface using four suckers on its anterior scolex. Migration of the proglottids to the gastrointestinal system lumen can lead to rare serious acute surgical conditions, such as acute appendicitis, Meckel’s diverticulitis, pancreatitis, cholecystitis, liver abscess, obstruction and perforation of the intestine, anastomotic leakage or appendicular stump dehiscence [4]. In previously published cases as well as the case we present, although it is difficult to establish the direct mechanism causing the complication, the etiological connection is highly probable. This entity can be diagnosed by detecting eggs or proglottids in the stool as early as approximately 3 months after infection [2]. The perianal region can be examined using a cellophane-tape swab to detect ova as well. Serologic tests are not helpful [3]. Infection by Teania saginata is preventable by improving sanitation and strict inspection of beef prior to sale. Beef should be cooked to 60°C for over 5 min before consumption [3]. praziquantel (5-10 mg/kg) is the drug of choice. Niclosamide is also used as it is cheap and available. Both drugs are very effective, simple to administer and comparatively free from side effects [7]. Surgery is indicated only for the treatment of complications [2].

## Conclusion

Post-appendectomy faecal fistula occur mostly when there is severe peri-appendicitis involving the base of the appendix as well as the adjoining caecal wall. An infestation with tapeworm is a very rare entity that should be considered in the diagnosis as the cause of Leakage from the appendiceal stump in endemic areas. It should be kept in mind as a very rare possible cause.
